# Description of a new species of the genus *Perissus* Chevrolat, 1863 (Coleoptera, Cerambycidae, Cerambycinae, Clytini) from China, with notes on its pollen loads composition

**DOI:** 10.3897/BDJ.14.e194990

**Published:** 2026-05-20

**Authors:** Luisa María Sandoval Pulgarín, Haiqiao Liang, Tao Zhang, Yujun Liu, Xiaoran Yang

**Affiliations:** 1 College of Biological Sciences and Biotechnology, Beijing Forestry University, Beijing 100083, China, Beijing, China College of Biological Sciences and Biotechnology, Beijing Forestry University, Beijing 100083, China Beijing China; 2 Jianfengling Tropical Rainforest National Park, Ledong, 572542, Hainan, China, Ledong, China Jianfengling Tropical Rainforest National Park, Ledong, 572542, Hainan, China Ledong China; 3 Hainan Jianfengling Forest Ecosystem National Field Science and Observation Station, Research Institute of Tropical Forestry, Guangzhou, 510520, China, Guangzhou, China Hainan Jianfengling Forest Ecosystem National Field Science and Observation Station, Research Institute of Tropical Forestry, Guangzhou, 510520, China Guangzhou China; 4 School of Forestry, Northeast Forestry University, Harbin 150040, China, Harbin, China School of Forestry, Northeast Forestry University, Harbin 150040, China Harbin China

**Keywords:** Cerambycinae, Hainan, longhorn beetle, new species, pollen, taxonomy

## Abstract

**Background:**

The genus *Perissus* belongs to the tribe Clytini, currently comprises 98 species and subspecies, widely distributed across the Palaearctic, Oriental and Australian Regions.

**New information:**

A new species, *Perissus jianfenglingensis* Sandoval, Liu & Yang **sp. nov**. [尖峰岭跗虎天牛] is described from Hainan Province, China, its morphological distinctions from related species are clarified, colour plates are illustrated and the characteristics of the species are provided. Additionally, a brief description and discussion of the pollen loads composition of the examined individuals were conducted.

## Introduction

The genus *Perissus* belongs to the tribe Clytini (Cerambycidae, Cerambycinae). It was established by Chevrolat in 1863, with *Perissus x-littera* Chevrolat, 1863 designated as its type species (Chevrolat 1863). The genus currently comprises 98 taxa and is widely distributed across the Palaearctic, Oriental and Australian Regions (Tavakilian and Chevillotte 2025). In China, 43 taxa have been recorded (Xie et al. 2025). Species of this genus can be distinguished from other genera within the tribe Clytini by the following combination of characters: frons without a longitudinal ridge, antennal insertions widely separated, antennomeres without apical spines and the first metatarsomere at least twice as long as the combined length of the second and third (Viktora and Tichý 2017).

During a recent survey of pollinating insects in Hainan Province, China, we identified a new species of *Perissus*. Pollen load analysis revealed that the examined individuals of this species carry pollen from only one type, which corresponds to that of *Castanopsis hystrix*. We describe and illustrate the new species herein and provide a brief discussion of this phenomenon.

## Materials and methods

### Study context and material collecting

**Figure 1.**
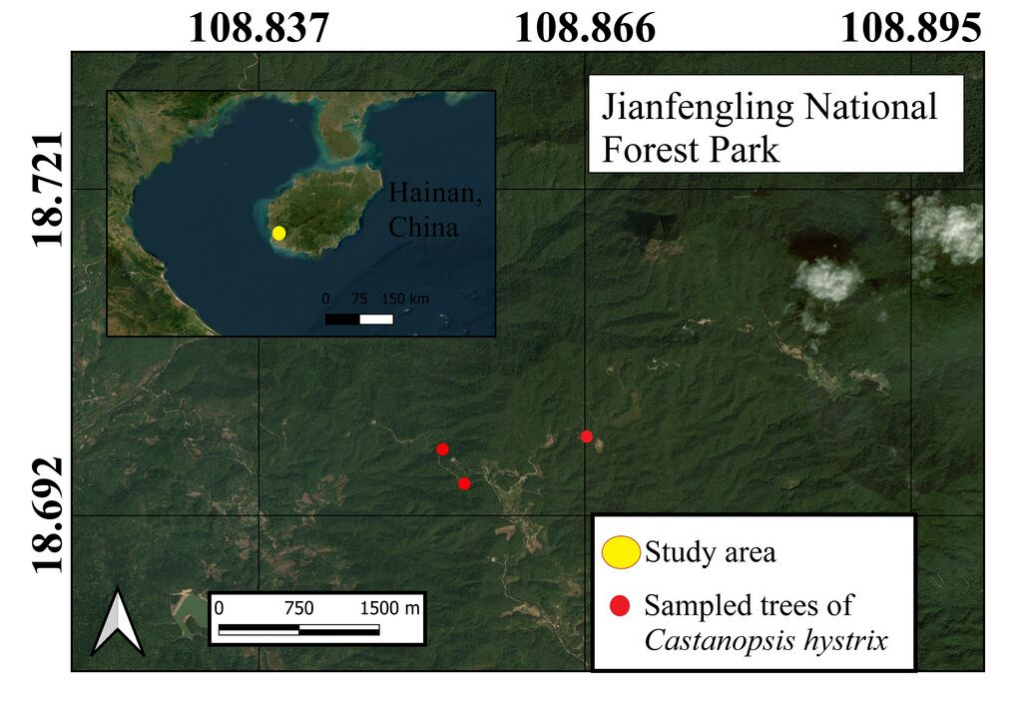
Collecting locality of *Perissus jianfenglingensis* sp. nov. from Hainan Island, China.

The insect individuals used in this study were collected as part of a larger research project investigating plant–beetle interactions on three Fagaceae species distributed along the Jianfengling Nature Reserve in Hainan, China (Fig. 1 and Fig. 2a). In that broader study, a total of 47 Coleoptera species (including 18 cerambycid species) were collected from the flowers of *Castanopsis hystrix* (Fig. 2b) using standardised methods over two consecutive years and their pollen loads were analysed. The five individuals of the species described in this manuscript were collected from three different *C. hystrix* trees distributed along an elevational gradient (552–775 m), ensuring spatial representativeness and reducing the possibility of local sampling bias. Detailed results of the broader study will be presented elsewhere; this study focuses on the taxonomic description and pollen load observations of this new species.

### Taxonomic methods

Preparation of Male Genitalia: Specimens were first softened. The male genitalia were then extracted from the abdominal apex using dissecting forceps. The removed genitalia were placed in a 10% potassium hydroxide (KOH) solution and digested at room temperature for 10 hours. Subsequently, they were cleaned and rinsed with distilled water under a stereomicroscope. After dissection, the separated parts were photographed and then stored in a small vial filled with glycerol.

Plates Preparation: Photographs of the adult specimens and genitalia were taken using a Canon EOS 6D Mark II camera, equipped with a Laowa 60 mm f/2.8 2× Ultra-Macro lens and a Laowa 25 mm f/2.8 2.5-5× Ultra Macro lens, respectively. The images were stacked using Zerene Stacker software to enhance depth of field and the final plates were compiled using Adobe Photoshop 2020.

### Reference pollen collection

Flowers of *C. hystrix* were collected directly from the same trees where the insect specimens were captured. Pollen from these flowers was used to create reference slides. A small amount of glycerine gelatine was warmed on a microscope slide and anthers were dissected and embedded in the gelatine. The slides were then sealed with transparent nail polish and labelled with the collection data. Acetylation was not performed to allow a direct morphological comparison with pollen extracted from the insects, which also would not undergo chemical treatment.The pollen description presented in the analysis was based on direct observation of flower samples collected from *C. hystrix* trees.

### Pollen extraction from insects

**Figure 3.**
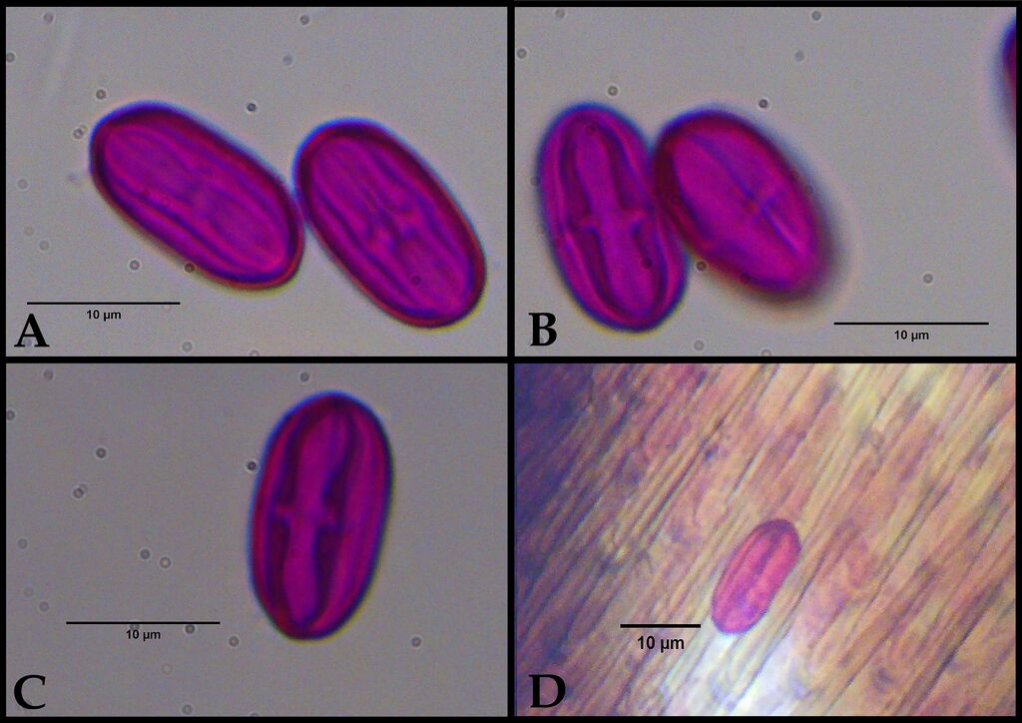
Reference pollen of **Castanopsis* hystrix* and a detail of one individual grain attached to the body of *Perissus jianfenglingensis* sp. nov. **A–C** Reference pollen taken from the *C. hystrix* flower; **D** Detail of a pollen grain of *C. hystrix* on the body (elytron) of *P. jianfenglingensis* sp.nov.

To avoid contamination, each of the five specimens was processed individually using a dedicated, disposable syringe pre-filled with glycerine gelatine. The pollen load was sampled by gently expressing a small amount of gelatine from the syringe tip and carefully rolling it over the entire body surface of the insect (including the head, thorax, abdomen and legs). This direct contact method allowed the gelatine to efficiently pick up the pollen grains adhered to the insect's integument. The pollen-laden gelatine was then applied directly on to a microscope slide, warmed to flatten the preparation and sealed with transparent nail polish. This technique provided a direct and contamination-free record of the pollen grains available for transport on each insect's body (Fig. 3D).

### Deposition of Materials

The insects material examined for this study is deposited in the following institutional and private collections: **CBJFC**: insect collection of Museum of Beijing Forestry University, Beijing, CHINA; **CNACRC**: insect collection of National Animal Collection Resource Center, Beijing, CHINA; **CPV**: collection of Pter Viktora, Kutna Hora, CZECH; All the pollen samples are deposited in collection of Beijing Forestry University, Beijing, CHINA.

## Taxon treatments

### Perissus jianfenglingensis

Sandoval, Liu & Yang sp. nov.

D15CFEDE-C59A-5DD6-8A1C-AD5F39BB7B4F

C585B0E3-C69E-4D41-92D7-E033F06ACF8F

#### Materials

- **Type status:** Holotype. **Occurrence:** catalogNumber: JFL2025CCCP21; recordedBy: Tao Zhang & Luisa Sandoval; sex: female; occurrenceID: 84ECB891-A9D8-5A76-B4A3-FE14A2A55BFF; **Taxon:** scientificNameID: *Perissus jianfenglingensis*; scientificName: *Perissus jianfenglingensis*; **Location:** country: China; countryCode: CN; stateProvince: Hainan; county: Ledong; municipality: Jianfeng Township; locality: Mt. Jianfengling National Natural Reserve [尖峰岭国家级自然保护区]; verbatimLocality: along trail ca. 4.7 km from entrance; verbatimElevation: 552 m; verbatimLatitude: 18°42'1"N; verbatimLongitude: 108°50'52"E; **Event:** samplingProtocol: sweep flower of *Castanopsis hystrix*; verbatimEventDate: 15 V 2025; **Record Level:** type: Holotype; institutionCode: Museum of Beijing Forestry University(CBJFC)
- **Type status:** Paratype. **Occurrence:** catalogNumber: JFL2025CCCP44; recordedBy: Haiqiao Liang, Tao Zhang & Luisa Sandoval; sex: male; occurrenceID: 646CE346-B95C-577B-B3FF-CEBB314F8266; **Taxon:** scientificNameID: *Perissus jianfenglingensis*; scientificName: *Perissus jianfenglingensis*; **Location:** country: China; countryCode: CN; stateProvince: Hainan; county: Ledong; municipality: Jianfeng Township; locality: Mt. Jianfengling National Natural Reserve [尖峰岭国家级自然保护区]; verbatimLocality: along trail ca. 5.6 km east from entrance; verbatimElevation: 637 m; verbatimLatitude: 18°41'43"N; verbatimLongitude: 108°51'18"E; **Event:** samplingProtocol: sweep flower of *Castanopsis hystrix*; verbatimEventDate: 21 V 2025; **Record Level:** type: Paratype; institutionCode: Museum of Beijing Forestry University (CBJFC)
- **Type status:** Paratype. **Occurrence:** catalogNumber: JFL2025CCCP47; recordedBy: Haiqiao Liang, Tao Zhang & Luisa Sandoval; sex: female; occurrenceID: 237DE3C8-9944-5FF4-82D1-9BFD6F1A2979; **Taxon:** scientificNameID: *Perissus jianfenglingensis*; scientificName: *Perissus jianfenglingensis*; **Location:** country: China; countryCode: CN; stateProvince: Hainan; county: Ledong; municipality: Jianfeng Township; locality: Mt. Jianfengling National Natural Reserve [尖峰岭国家级自然保护区]; verbatimLocality: along trail ca. 5.6 km east from entrance; verbatimElevation: 637 m; verbatimLatitude: 18°41'43"N; verbatimLongitude: 108°51'18"E; **Event:** samplingProtocol: sweep flower of *Castanopsis hystrix*; verbatimEventDate: 21 V 2025; **Record Level:** type: Paratype; institutionCode: Museum of Beijing Forestry University, Beijing, China(CBJFC)
- **Type status:** Paratype. **Occurrence:** catalogNumber: JFL2025CCCP11; recordedBy: Haiqiao Liang, Tao Zhang & Luisa Sandoval; sex: female; occurrenceID: 9EEB6710-9DAA-55E7-8727-017DC0714DF6; **Taxon:** scientificNameID: *Perissus jianfenglingensis*; scientificName: *Perissus jianfenglingensis*; **Location:** country: China; countryCode: CN; stateProvince: Hainan; county: Ledong; municipality: Jianfeng Township; locality: Mt. Jianfengling National Natural Reserve [尖峰岭国家级自然保护区]; verbatimLocality: along trail ca. 5.6 km east from entrance; verbatimElevation: 637 m; verbatimLatitude: 18°41'43"N; verbatimLongitude: 108°51'18"E; **Event:** samplingProtocol: sweep flower of *Castanopsis hystrix*; verbatimEventDate: 21 V 2025; **Record Level:** type: Paratype; institutionCode: Museum of Beijing Forestry University, Beijing, China(CBJFC)
- **Type status:** Paratype. **Occurrence:** catalogNumber: JFL2024CCCP112; recordedBy: Haiqiao Liang, Tao Zhang & Luisa Sandoval; sex: female; occurrenceID: C042D4AA-4D57-5289-BAA4-955D9FA90C05; **Taxon:** scientificNameID: *Perissus jianfenglingensis*; scientificName: *Perissus jianfenglingensis*; **Location:** country: China; countryCode: CN; stateProvince: Hainan; county: Ledong; municipality: Jianfeng Township; locality: Mt. Jianfengling National Natural Reserve [尖峰岭国家级自然保护区]; verbatimLocality: along trail ca. 7.2 km east from entrance; verbatimElevation: 775 m; verbatimLatitude: 18°41'58"N; verbatimLongitude: 108°51'57"E; **Event:** samplingProtocol: sweep flower of *Castanopsis hystrix*; verbatimEventDate: 2 VI 2024; **Record Level:** type: Paratype; institutionCode: National Animal Collection Resource Center, Beijing, China(CNACRC)

#### Description

**Figure 4.**
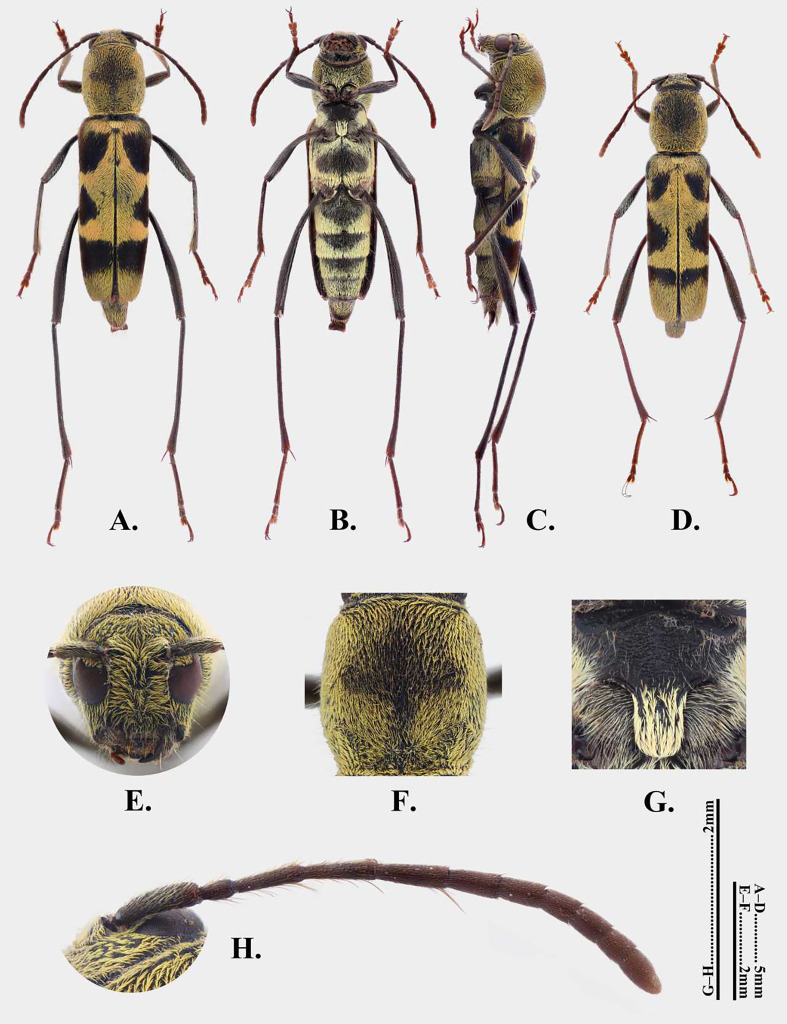
*Perissus jianfenglingensis* sp. nov. **A–C, E–H** female holotype; **D** male paratype. **A–D** habitus. **A, D** dorsal view; **B** lateral view; **C** ventral view. **E–H** Detailed views of various parts. **E** head in frontal view; **F** pronotum; **G** mesoventrite; **H** antenna.

**Female**. Body length 11.5–12.4 mm, humeral width 3.1–3.7 mm (11.5 mm and 3.1 mm in Holotype). Body black to blackish-brown; head, thorax and most of abdomen covered with yellow pubescence; pubescence on elytra forming bands; colouration of setae on legs variable (Fig. 4A–C).

**Head**: Approximately as wide as anterior margin of pronotum, with irregular punctures and depressions; ground colour black, covered with yellow pubescence. Frons flat, width equal to height, without a median ridge (Fig. 4E). Eyes large, lower lobe about as long as wide, slightly longer than gena.

**Antennae**: Antennae short, only slightly exceeding elytral base, gradually thickening from antennomere III, finely punctate, mostly covered with brownish setae; antennomeres I–IV with long, stout erect setae, V–XI with fine, dense short pubescence. Outer side of scape covered with pale yellow setae; inner side of antennomeres II–V with brownish fringe, setae longer at apices and gathered to form a spine-like projection (Fig. 4H); antennomeres III–V subequal in length, slightly shorter than scape, approximately twice as long as pedicel; subsequent segments shorter; segment VI about as long as terminal segment, slightly longer than VII; VIII–X subequal in length, slightly shorter than VII.

**Pronotum**: Cylindrical, longer than wide, widest point approximately as wide as elytral base, with dense, coarse punctures; ground colour black, mostly covered with yellow pubescence, setae denser on anterior and lateral margins. Pronotal disc slightly convex, with a somewhat rhomboid area covered with fine, yellowish-brown pubescence on disc and a curved area near base covered with short, yellow pubescence; these two areas slightly connected (Fig. 4F). Prosternum with paler setae; procoxal cavities open posteriorly. Scutellum semicircular, covered with yellow pubescence.

**Elytra**: Subrectangular, approximately three times as long as wide, with fine punctures; sides parallel; apex obliquely truncate; ground colour black; areas reddish-brown including: spots on both sides of scutellum and a nearly circular spot on lateral side near apical 2/3, as well as an oblique, arcuate band extending from below scutellum to lateral margin near basal 1/3. Remaining surface mostly with yellow pubescence forming bands and small areas with pale short pubescence forming black maculae. Position and shape of black maculae are as follows: a subsemicircular spot obliquely below scutellum; a triangular spot directed towards suture in middle of elytra; a subtrapezoidal spot near elytral apex.

**Venter**: Mostly with fine punctures, covered with pale yellow pubescence; disc of metaventrite and anterior margins of ventrites I–III with fine, yellowish-brown pubescence. Mesoventrite nearly glabrous, with irregular punctures and wrinkles; mesoventral process tongue-shaped, covered with pale yellow pubescence (Fig. 4G).

**Legs**: Slender, blackish-brown, paler near apices, with fine punctures; setation as follows: profemora, mesofemora and most of tibiae with pale pubescence; inner side of tibiae with short brown setae, with yellowish-brown setal brushes near apices; metafemora and metatibiae mostly with brownish pubescence, only outer side of femora near base with sparse pale pubescence; tarsi covered with yellowish-brown short pubescence. Claws simple. Protibiae and mesotibiae with one short apical spur; metatibiae with two apical spurs, one long and one short. First metatarsomere approximately three times the combined length of the second and third.

**Male**. Body length 10.5 mm, humeral width 2.7 mm. Generally similar to female, but pattern formed by pubescence slightly variable (Fig. 4D).

**Figure 5.**
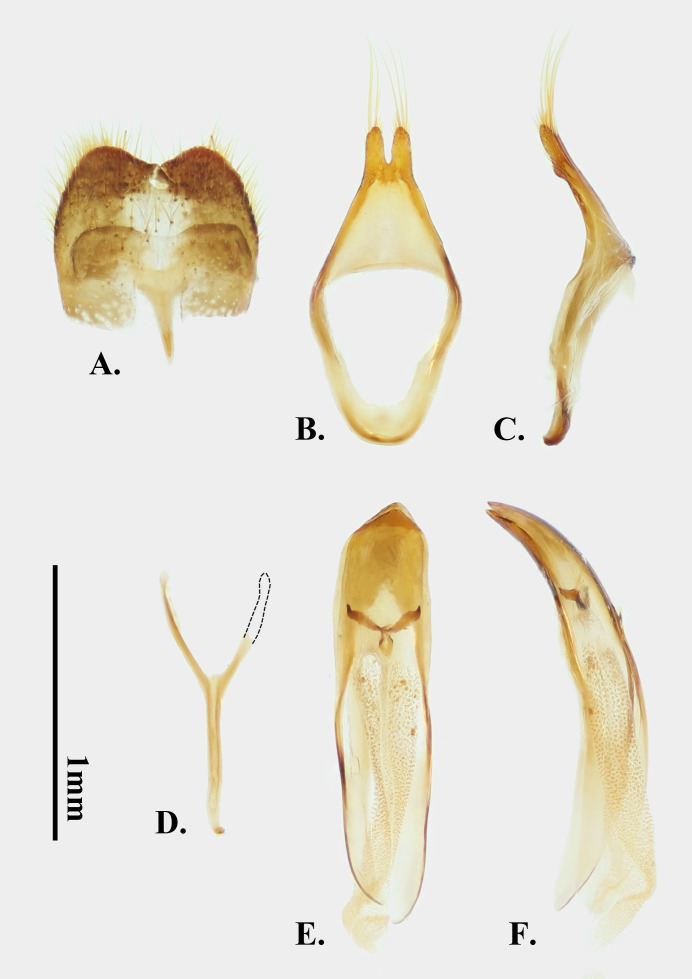
Male terminalia of *Perissus jianfenglingensis* sp. nov. **A** tergite & sternite VIII; **B, C** tegmen; **D** sternite IX; **E, F** median lobe. **A** dorsal view; **B, D, E** ventral view; **C, F** lateral view.

**Figure 6.**
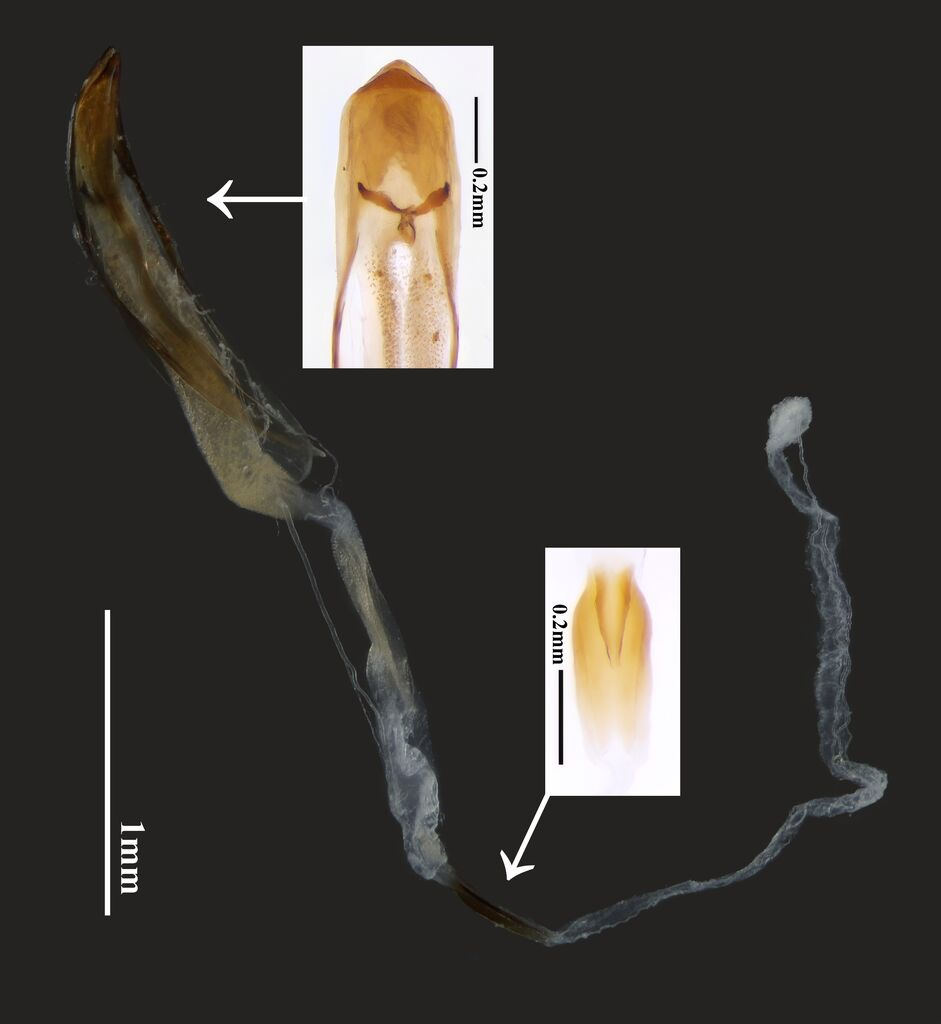
Median lobe with endophallus of *Perissus jianfenglingensis* sp. nov. (with close-up of basal sclerite and median sclerotised ring).

**Male terminalia**: Tergite VIII as Fig. 5A, somewhat trapezoidal, concave at apical margin, with irregular depressions near apex, with relatively short setae. Spiculum gastrale of sternite IX (Fig. 5D) with stem slightly longer than arms. Tegmen (Fig. 5B and C) relatively broad; ringed part slightly thickened; parameres short and small, approximately 1/6 the length of tegmen and with apices rounded and blunt, bearing about six long setae. Median lobe (Fig. 5E and F) robust, slightly curved in lateral view; ventral and dorsal plates not sharply pointed at apex, apices aligned; basal struts diverging from basal 1/3 of median lobe; median struts well developed. Endophallus, in uneverted and uninflated state, approximately 3.5 times as long as median lobe, with claw-like sclerites at base; median phallomere covered with small granules, with one elongate, narrow sclerotised ring at about mid-length. Structure as Fig. 6.

#### Diagnosis

The yellow-and-black colouration of Perissus jianfenglingensis sp. nov. is quite common within the tribe Clytini, but the genus Perissus can be distinguished from the genera Xylotrechus and Clytus by the absence of a longitudinal ridge on the frons and by the first metatarsomere being three times the length of the combined length of the second and third segments. In the genus *Perissus*, the new species is most similar in appearance to *P. tunicatus* Viktora & Liu, 2018 (Fig. 7a) and *P. lubosi* Viktora, 2020 (Fig. 7b) (Viktora and Liu 2018, Viktora 2020), but can be distinguished by the following characteristics: in terms of pubescence colour, the head and pronotum of the new species are similar in colour to the yellow bands on the elytra, whereas those of *P. lubosi* and *P. tunicatus* are significantly darker than the yellow bands on the elytra. In addition, the disc of the pronotum of the new species bears a nearly ginkgo leaf‑shaped area covered with fine yellowish‑brown pubescence, a feature absent in the other two species. On the antennae, the antennae of *P. lubosi* are distinctly covered with white pubescence, while those of the new species are not; the antennae of *P. tunicatus* are longer than those of the new species. On the pronotum, the new species is distinctly more square, while the other two are more rounded. On the elytra, the reddish-brown area at the base of the elytra of *P. lubosi* is larger, while *P. tunicatus* lacks red colouration at the base; the second pair of black spots on the elytra of the new species are not connected to form a band, whereas, in the other two species, they are connected to form a band. In addition, the currently known distribution ranges of these three species are all very narrow and they are located far apart from each other.

#### Etymology

The specific epithet is derived from its type locality.

#### Distribution

Hainan Province, China.

## Analysis

### Pollen load analysis

The individuals of *Perissus jianfenglingensis* sp. nov. exhibited a pollen load consisting of a single pollen type (corresponding to *C. hystrix* pollen type) (Fig. 3D).

The pollen of *C. hystrix* is characterised by monad, isopolar, small-sized grains. In polar view, the outline is triangular with rounded margins. In equatorial view, the grains are oblate-spheroidal. They are tricolporate, with long colpi and lalongate endo-apertures. The pollen grains are small, ranging from 15 to 20 micrometres in size (Fig. 3A-C).

The total pollen count per individual varied amongst the five examined specimens of *Perissus jianfenglingensis* sp. nov., with 81, 25, 207, 41 and 29 grains recorded (Table 1).

## Discussion

This study integrates taxonomic and palynological techniques to describe a new cerambycid species and to characterise the pollen load composition of the captured specimens. The results concerning the pollen load composition of *P. jianfenglingensis* sp. nov. are particularly interesting, as the sampled individuals were collected from different *Castanopsis hystrix* trees distributed across distinct localities. Moreover, during the collection period, several other plant species co‑flowered with *C. hystrix* in the surrounding area, including *Albizia attopeuensis* (Pierre) Nielsen, *Gmelina chinensis* Benth., *Hydnocarpus hainanensis* (Merr.) Sleumer, *Dimocarpus longan* Lour. and *Elaeocarpus chinensis* Hook. ex Benth. This reduces the possibility that limited floral resources alone could explain the exclusive pollen loads documented. Therefore, the fact that these individuals carried exclusively *C. hystrix* pollen suggests that the observed pattern cannot be attributed solely to local floral availability or sampling bias. Although confirmation would require additional sampling, this finding supports the interpretation of a potential specialised association.

Previous studies have reported insect visitors on Fagaceae flowers, including members of the Diptera and Coleoptera (Petit and Larue 2022, Larue and Petit 2024, Chen 2025). In some cases, such as on certain *Castanea* trees, Cerambycidae species have also been documented as part of these visitor assemblages (Larue 2021, Jacobson and Pilkey 2024). Our findings represent the first record of a cerambycid species closely associated with *C. hystrix* trees.

The detection of exclusive pollen loads on individuals of *P. jianfenglingensis* sp. nov., combined with the fact that all specimens were collected from different localities (covering various local conditions and an altitudinal gradient) and during two distinct flowering periods (2024–2025), suggests a preliminary observation of a potential specialised relationship involving this species. To further explore the nature of this possible interaction, future research would benefit from incorporating a larger sample size and encompassing multiple seasons and sites. Additionally, direct observations of flower‑visiting behaviour, along with studies on the life cycle of this insect, would be valuable in determining the ecological significance of this association. Consequently, the results presented herein regarding pollen load composition provide only an initial insight into a possible pattern.

### Conclusions

This study provides a taxonomic description of the new cerambycid species *P. jianfenglingensis* sp. nov. from Hainan, China, complemented by observations on its pollen load composition. It was found that individuals of *P. jianfenglingensis* sp. nov. carried pollen exclusively from *C. hystrix*. These findings together suggest a possible specialised interaction between this new cerambycid species and *C. hystrix* trees.

This case illustrates how integrating taxonomic and palynological approaches can provide preliminary insights into the ecological roles of understudied insect groups.

## Supplementary Material

XML Treatment for Perissus jianfenglingensis

## Figures and Tables

**Figure 2a.**
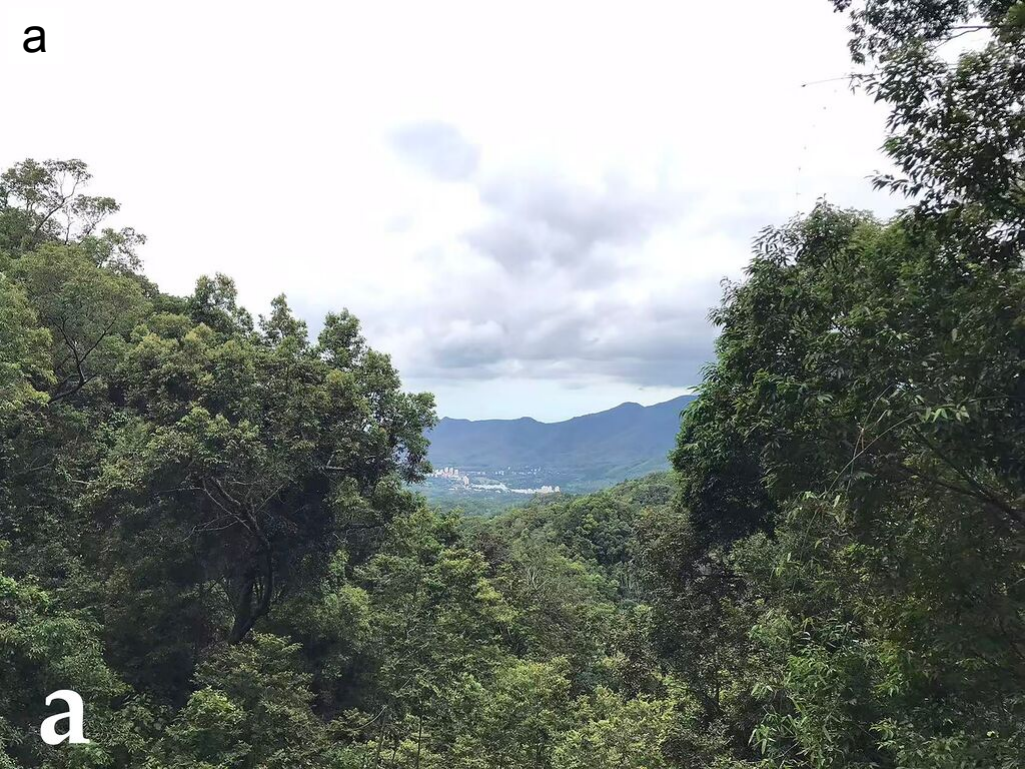
General environment where the trees are distrbuted inside the Jianfengling National Nature Reserve;

**Figure 2b.**
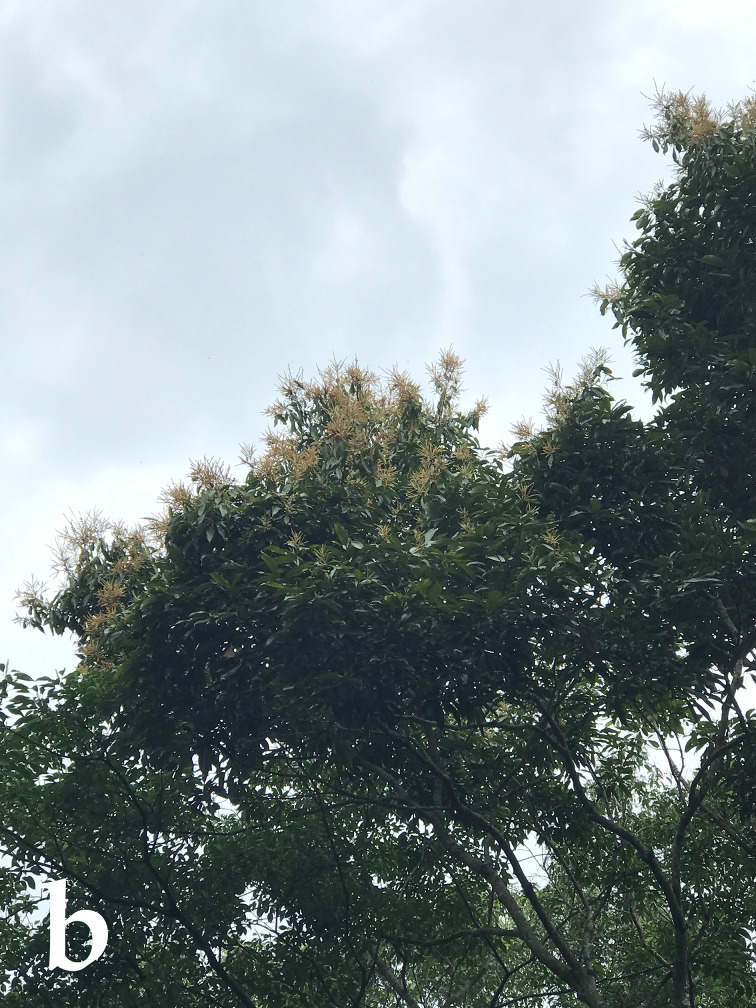
Flowers of **Castanopsis* hystrix* where the insect was collected.

**Figure 7a.**
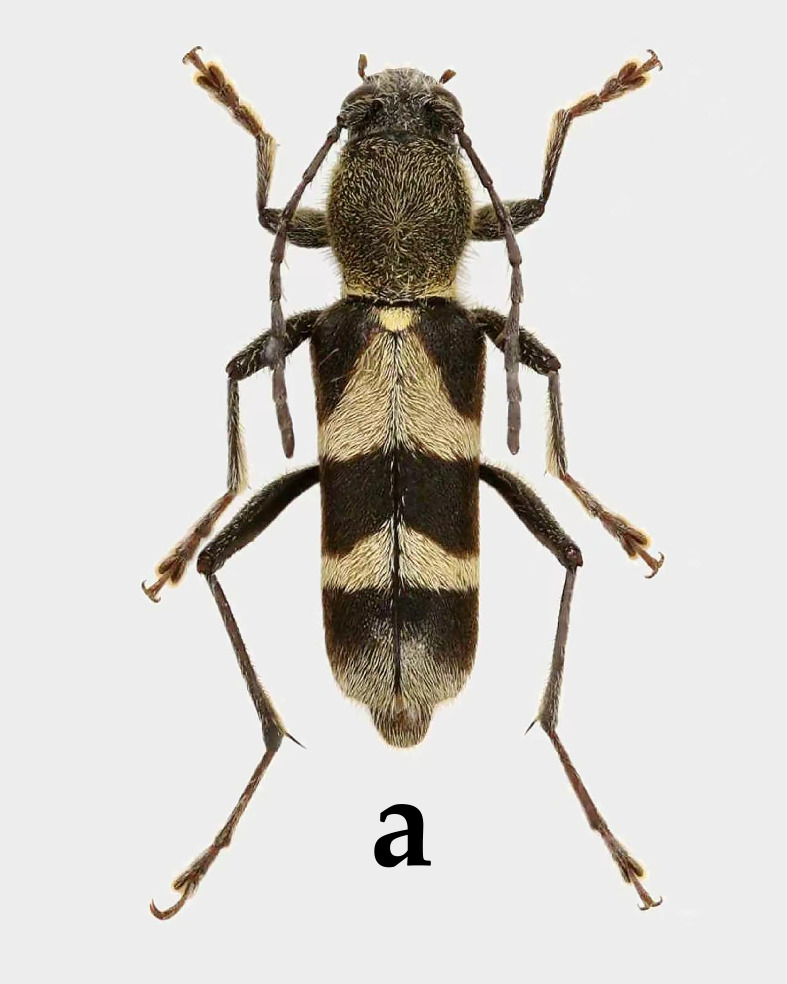
*Perissus tunicatus* Viktora & Liu, 2018, holotype, male;

**Figure 7b.**
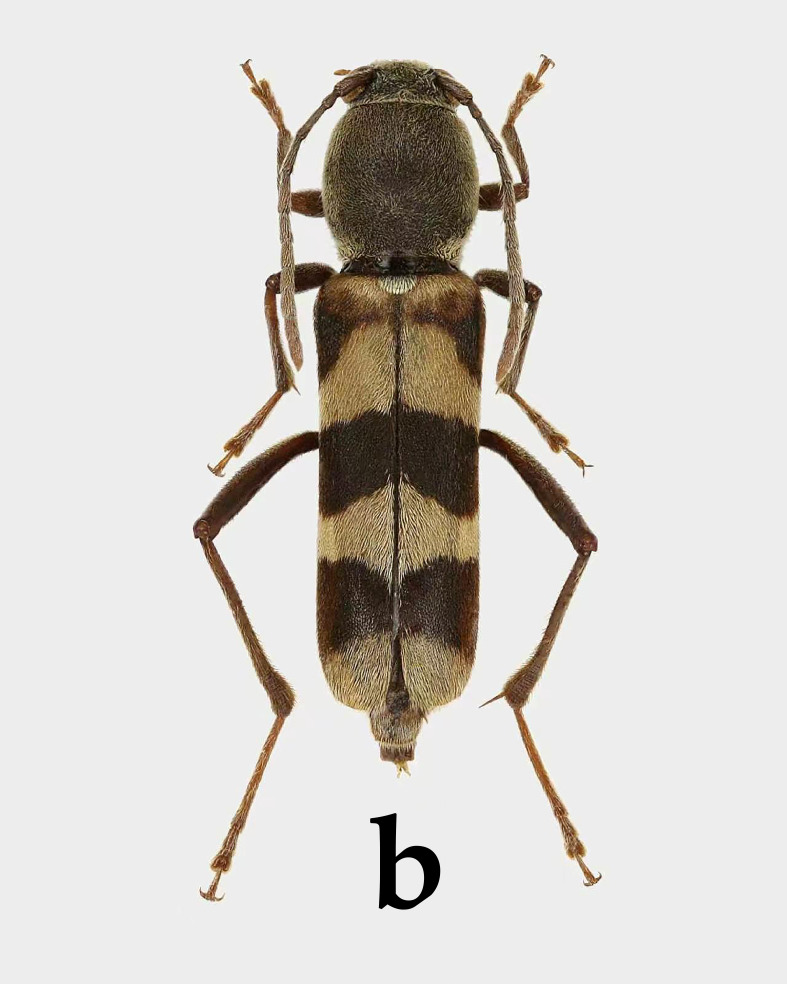
*Perissus lubosi* Viktora, 2020, Holotype, female.

**Table 1.** Results of the pollen load analysis for the five specimens of *Perissus jianfenglingensis* sp. nov.

**Specimen examined**	**Total number of pollen grains on the sample**	**Percentage of *C. hystrix* pollen in the load**
JFL2025CCCP212	81 grains	100%
JFL2025CCCP444	25 grains	100%
JFL2025CCCP47	207 grains	100%
JFL2025CCCP11	41 grains	100%
JFL2024CCCP112	29 grains	100%
